# Equity financing intention of elderly care enterprises: Influence of institutional logic and operation mode

**DOI:** 10.3389/fpubh.2022.811876

**Published:** 2022-10-18

**Authors:** Yiqi Zhao, Dong Wei, Xianfeng Zhao, Xianglan Dong, Luzhi Guo

**Affiliations:** ^1^School of Management, Shijiazhuang Tiedao University, Shijiazhuang, China; ^2^School of Management, Hebei GEO University, Shijiazhuang, China

**Keywords:** institutional logic, operating mode, elderly care enterprises, equity financing intention, business performance

## Abstract

The elderly population in China is expected to exceed 300 million and enter the stage of moderate aging during the 14th Five-Year plan period from 2021 to 2025. From the sustainable development perspective of elderly care enterprises, the supply of elderly care services would be unsustainable if enterprises suffer long-term losses. In the latter pursuit of high profits, the burden on consumers will increase. Equity financing of these enterprises is the key to achieving high-quality transformation and development by considering economic and social benefits. This study considers 20 well-known China-based elderly care enterprises as the research object. It uses a fuzzy set to explore system logic, operation mode, management performance, and attitude of elderly care enterprises toward investment through the qualitative comparative analysis method. The causal relationship between them is clarified—because, before the endowment enterprise equity financing intention of China, it is important to explore the effective path of equity financing of endowment enterprises. In the past, this helped Chinese elderly care enterprises actively cope with the trend of population aging, meet the needs of diversified and multi-level elderly care services, establish a sustainable development mode, and achieve high-quality transformation and development. The results show that (1) the operating performance of elderly care enterprises under the mode of public construction and private operation is poor; (2) elderly care enterprises driven by public welfare logic are more likely to achieve higher business performance, and (3) elderly care enterprises driven by business logic are more willing to introduce investment when they have made profits.

## Introduction

China had 267.36 million people aged 60 years and older in 2021, accounting for 18.9% of the population of the country. The Ministry of Human Resources and Social Security has predicted that during the 14th five-year plan period, the elderly population of China will exceed 300 million and enter the stage of moderate aging. Currently, the social scale of the aging population of China is large, the speed of aging is fast, and the endowment of urban and rural development is unbalanced. State Assets Report found that development of the elderly care industry in China lags behind the aging trend and the aging population market demand. Therefore, the elderly care industry must take the development path of scale, chain, and informatization.

From the sustainable development perspective of elderly care enterprises, the supply of elderly care services will be unsustainable if enterprises suffer long-term losses. The burden on consumers will increase if these enterprises continue pursuing high profits. The equity financing intention of enterprises is the key to achieving high-quality transformation and development by considering economic and social benefits. The system logic of elderly care enterprise development includes business and social welfare logic, that is, its pursuit of economic and social benefits.

In particular, Pache and Santos (1) pointed out that the logic of business and social good are independent and differ significantly in goals, means, organizational forms, and sources of professional legitimacy. Business logic emphasizes efficiency, market rules, and the pursuit of profit maximization. By contrast, social public welfare logic emphasizes democracy and autonomy, the pursuit of solving social problems, and creating social values. Existing studies lack a panoramic investigation of the composition dimension and division type of the multi-institutional logic of elderly care enterprises. Future research should enrich the investigation of the institutional logic management model, its constitutive dimension, and the classification type of social enterprises, especially to identify the possible unique dimensions and characteristics of the institutional logic management model of social enterprises in the Chinese context (2). There is a lack of a holistic theoretical framework for the leading factors and subsequent results of the logical management mode of elderly care institutions (3). The context of existing research regarding the multi-institutional logic of elderly care institutions is mostly based on Western countries (4), and the research on the internal multi-institutional logic of elderly care enterprises under the background of high-quality development in China is relatively lacking.

Driven by different sets of institutional logic, elderly care enterprises choose different operation modes, such as wholly owned self-funding, cooperative investment, and crowdfunding modes. The system logic and business model will affect elderly care enterprise business performance and determine their equity financing intention.

The problem of the aging population is becoming increasingly severe. The standardization, scale, and expanding chain of elderly care enterprises need a large amount of capital support to improve the elderly care service supply system (5, 6). Existing studies have paid little attention to the antecedents affecting the equity financing intention of such enterprises.

It would be helpful for Chinese elderly care enterprises to respond actively to this aging trend. It would enable them to meet the diversified and multi-level demand for elderly care services, establish a sustainable development mode, and realize high-quality transformation and development by clarifying the esthetics of equity financing intention and exploring an effective path of equity financing.

Therefore, this study considers 20 well-known endowment enterprises as the research object and uses a fuzzy qualitative comparative analysis method to explore the causal relationship among system logic, operation mode, management performance, and elderly care business attitude to investment. It is expected to introduce suitable investors for pension enterprises in Hebei Province, steadily expand the enterprise scale, and improve the supply system and their quality of service. This can effectively meet the diversified and multi-level needs of elderly care services and provide them with a theoretical basis and decision-making reference.

## Literature review

### Operating mode and performance of elderly care enterprises

The service mode of elderly care enterprises includes institutional, community, and home elderly care services. The operation mode of elderly care enterprises can be divided into wholly-owned self-financing, cooperative investment, real estate investment trust (REIT), trust investment, and build–operate–transfer (BOT) modes. The wholly owned self-financing mode mainly refers to the operation of elderly care enterprises that expands their funds through existing funds or other financing channels (7). The public construction and private construction mode is an important way to stimulate the vitality of public elderly care institutions and promote the participation of social forces. Public construction and private operation entail the government abandoning all high-consumption and low-efficiency management systems and operation mechanisms while building old-age service institutions. According to the development idea regarding the separation of management and operation, the government funds and bids social organizations or service groups to manage and operate the institutions, while it is responsible for their management and supervision. Public and private elderly care institutions are non-profit. Even if non-governmental organizations or enterprises are introduced for management and operation, the institution still cannot make profits, and the welfare and public welfare of the institution should be reflected in the occupancy price and qualification (4). Public and private elderly care institutions should operate according to marketization. Apart from public elderly care institutions managed by the government, public and private elderly care institutions need to achieve self-sustainable development through operation and management. The government provides construction-related service, bed, and operation subsidies according to relevant standards to support the sustainable development of public and private elderly care institutions. The third is the crowdfunding mode. Early interview materials found that elderly care enterprises were established by crowdfunding with their partners. Accordingly, this study will explore the impact of service and operation modes on elderly care enterprise business performance.

### Institutional logic of elderly care enterprises and performance

There are three sets of system logic for social enterprises in China: business, social welfare, and government logic (8). Social welfare logic aims to find social pain points, solve social problems, create social value, and promote social innovation and change. The means to achieve the logical goal of social good include responsibility, community autonomy, volunteers, and public participation. The aim is to follow the business logic, whose goal is to survive, make profits, and stimulate growth. The means to achieve business logic objectives include business capability, business model, technological innovation, customer orientation, and resource acquisition and utilization. Most elderly care companies pursue a dual goal, achieve financial sustainability, and create social value, which is significantly different from commercial enterprises that simply pursue profit maximization, and non-profit organizations that focus on creating social value. In addition, the realization of elderly care enterprise organizational goals is mission- and market-driven, which should consider the needs of customers and the appeals of beneficiaries. The theoretical discussion and empirical study on how institutional logic affects organizational performance will open a new research path for studying elderly care enterprises (3). The impact of institutional logic on performance is a dynamic and long-term process, closely related to internal tension. It corresponds to management strategies and governance structures faced by elderly care enterprises (9). This study will reveal the impact of institutional logic on the business performance of elderly care enterprises. Most founders of such enterprises set them out of love and the consciousness of serving society. To continuously provide services to the elderly, the business logic of ensuring the long-term operation of such enterprises will also affect those who operate them (10). Therefore, most elderly care enterprises have multiple sets of institutional logic. Social logic and business logic coexist. The institutional logic of all—the wholly owned self-funded elderly care enterprises, public construction, private elderly care enterprises, and crowdfunding enterprises—varies.

### Performance of elderly care enterprises and their equity financing intention

Capital is profit-driven. The appeal of self-interest may also push the focus of external investment too much on its short-term benefits. External investment can provide external resources for elderly care enterprises. From the concrete influence mechanism, external investment can provide enterprises with direct capital flow, indirect information flow, and technology flow. First, capital flow provision is a direct mechanism for venture capital to help elderly care enterprises. Start-up elderly care enterprises have uncertain profit prospects, insufficient collateral, and an unstable profit flow, which make it difficult to obtain indirect financing from banks, thereby causing difficulties in carrying out elderly care services. Investment institutions have professional experience in project management and market demand. They can provide enterprises with technical resources oriented to project operation, management, and development. This can reduce the management cost of enterprises to a certain extent (11). The behavior of external investment chasing short-term profits is not conducive to the long-term sustainable development of elderly care enterprises. Some scholars have pointed out that external investment in enterprises does not actively seek to serve society but to pursue profits (12). The influence of external investment on corporate social benefits is not completely correlated and has a crowding-out effect on corporate social benefits (13). Profitable elderly care enterprises easily reach agreements with investors. The investors ask elderly care enterprises to adjust the management strategy; their management rights and decision-making power will be affected if they lose. In improving the supply system, elderly care enterprise standardization, scale, and chain expansion require a large amount of capital support (5, 6). Existing studies have paid little attention to the factors affecting the investment introduced by elderly care enterprises.

Corporate earnings may also influence the attitude of an endowment enterprise toward investment. The investor is bound to the enterprise management concept and management pattern question if a loss-making state investment introduces the endowment enterprise. Under the trend of short-term profit goals, investors are likely to reduce reversed transmission endowment enterprise long-term strategic investment to give up for service to mid-level income of the elderly. However, the low-margin business is at odds with the social consciousness of elderly care companies (11, 14). Based on this, this study has explored the relationship between the operating performance of elderly care enterprises and their investment attitude.

## Methods

**Figure 1:**
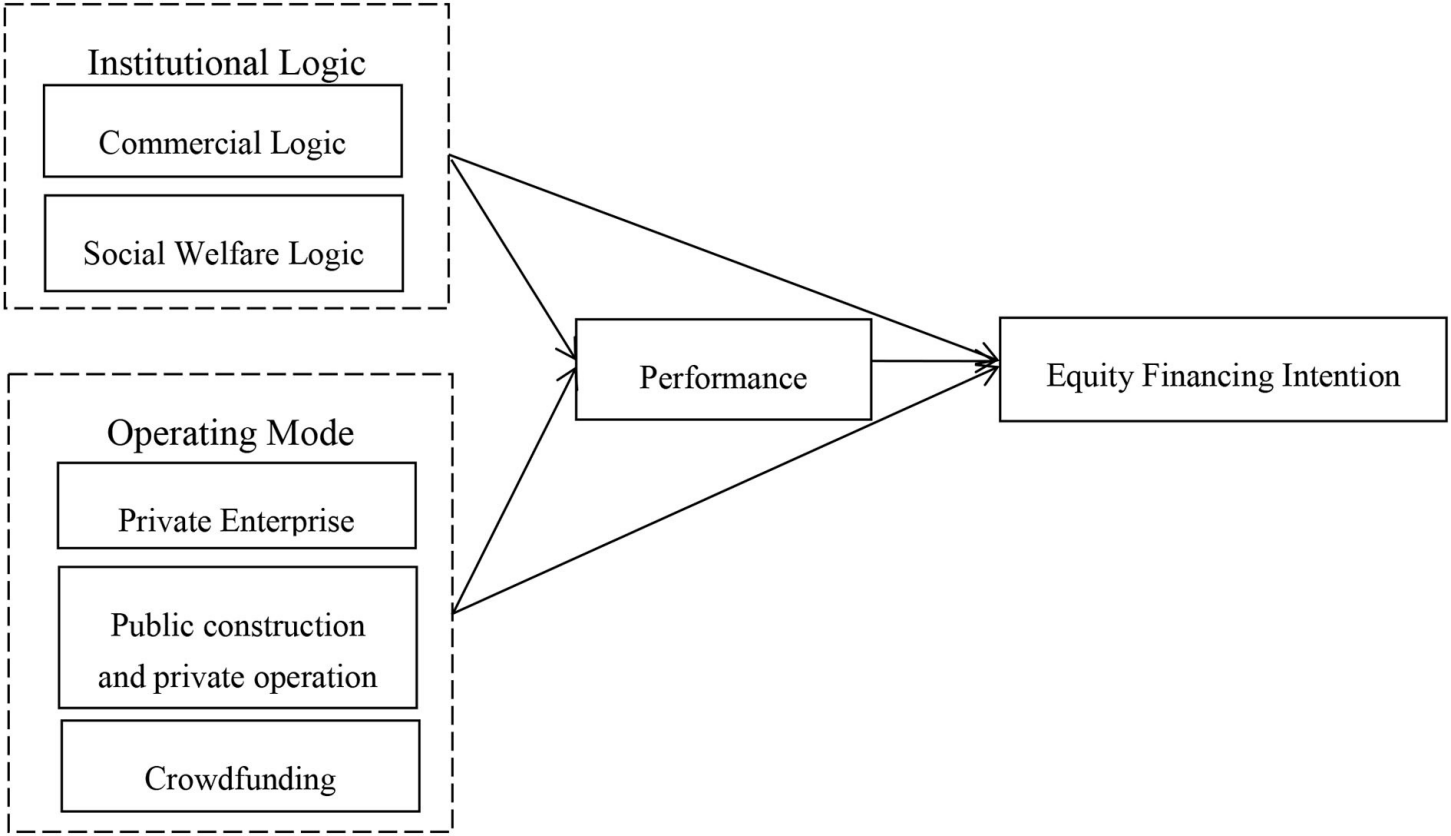
Research framework.

The study selected 20 elderly care enterprises involved in the interview program of the founder of a well-known elderly care enterprise. Respect the elderly in your family as well as the elderly of other families. As the aging trend is increasingly obvious, the demand for elderly care is gradually increasing, and a well-known old-age care dialogue program came into being. We-Media elderly care dialogue program, jointly created by a well-known health newspaper and senior scholars in health and elderly care, is the first large-scale program in China. From 2017 to 2020, the program was conducted to interview 30 elderly care enterprises. The study dealt with the equity financing intention of the founders of elderly care enterprises. After evaluating the interview data of all 30 programs, the researcher selected 20 elderly care companies whose interview content involved the founder's intention of equity financing as the research object. The 10 elderly care companies whose other interview content did not involve relevant topics were not selected. The program interview video, the official website of related elderly care companies, and the news were the sources of information. The research framework is shown in Figure 1.

The method of data coding and qualitative comparative analysis of fuzzy sets was used to study the causal relationship between variables. This study follows the data analysis procedures recommended by Strauss and Corbin (15) to carry out data coding such as operating mode, institutional logic, performance, and equity financing intention of elderly care enterprises. This was carried out to ensure the accuracy and verifiability of the research conclusions. “Yes” was marked as “ 1” and “no” as “0”.

First, a coding team was set up. To avoid the influence of researchers' subjective bias on coding and to reduce error in research results, one author from the financial management department was selected as the group leader; two authors from the technical economy and management and strategic management departments formed the coding team. The group leader provided theoretical and technical training for the other two group members. Subsequently, each was responsible for the trial coding of a case object. They discussed results to find problems in the coding process and recommended solutions. Consequently, the team members did the coding work on the case objects. They discussed their contrary opinions until the coding results were consistent.

Second, a coding database was built. Excel was used to create a form for each case object to record the detailed coding process. The modification steps in the process were recorded.

Finally, the qualitative comparative analysis of fuzzy sets was divided into three steps. The first found the correspondence between the vector space angle defined by the fuzzy set and rows in the truth table. The second was to evaluate the distribution of cases in various combinations of conditions. The third was to evaluate the consistency of the result's fuzzy subset.

This study followed the research of Rihoux and Ragin (16) to conduct fsQCA research. Before condition configuration analysis, the necessity and adequacy of condition variables should be analyzed. A condition is always present in the presence of an outcome, and this condition is a necessary condition for the existence of the outcome. An important measure of necessity is consistency, which is generally considered to have a minimum score of 0.8. The original coverage represents the proportion of cases in which the combination of conditions leads to the occurrence of the result in all cases. Unique coverage represents the percentage of cases in which this combination of conditions causes an outcome to occur, while no other combination of conditions causes an outcome to occur.

## Results

Table 1 presents the deduced complex solution, illustrating the causal formula (i.e., sufficient conditions), which leads to high membership in the five outcome conditions. The complex solution is contrary to the concise and intermediate solutions, and there is no simplifying assumption (17). After calculating the consistency scores of all possible complex causal combinations leading to these six outcome conditions, they were compared with the usual 0.80 cutoff consistency score. Combinations with agreement scores above this threshold would remain in the final solution. Table 1 shows that all four models (solutions) provide much information. As Woodside (18) pointed out, most agreement values were higher than 0.75, and most coverage values were between 0.10 and 0.65.

**Table 1:** Intermediate solution for the introduction of investment by elderly care enterprises.

**Intermediate solution**	**Raw coverage**	**Unique coverage**	**Consistency**
**Performance**
Model 1: performance = f(private enterprise, public construction and private operation, Crowdfunding)			
Private enterprise*~public construction and private operation*~Crowdfunding	0.647059	0.647059	0.785714
~private enterprise*~public construction and private operation*Crowdfunding	0.117647	0.117647	1.000000
Solution coverage: 0.764706; Solution consistency: 0.8125			
Frequency cutoff: 2; consistency cutoff: 0.785714			
**Performance**
Model 2:performance=f(Commercial logic, Social welfare logic)			
Social welfare logic	1.000000	1.000000	0.850000
Solution coverage:1; Solution consistency: 0.85			
frequency cutoff: 7; consistency cutoff: 0.769231			
**Equity financing intention**
Model 3:equity financing intention = f(Commercial logic, Social welfare logic, private enterprise, public construction and private operation, Performance)			
Commercial logic*~public construction and private operation*Performance	0.666667	0.466667	0.769231
Commercial logic*~Social welfare logic*private enterprise*Performance	0.333333	0.133333	0.833333
~Commercial logic*Social welfare logic*~private enterprise*public construction and private operation*~Performance	0.066667	0.066667	1.000000
Solution coverage:0.866667; Solution consistency: 0.8125			
frequency cutoff: 1; consistency cutoff: 0.714286			
**Equity financing intention**
Model 4:equity financing intention = f(Commercial logic, Social welfare logic, Crowdfunding, Performance)			
Commercial logic*~Social welfare logic*Performance	0.4	0.333333	0.857143
Commercial logic*Crowdfunding*Performance	0.133333	0.0666667	1.000000
Solution coverage: 0.466667; Solution consistency: 0.875			
Frequency cutoff: 1; consistency cutoff: 0.833333			

### Operating mode and performance of elderly care enterprises

From their operating mode, there are two ways to realize profits for elderly care enterprises. The first path shows that the operating mode is only for private enterprises, which is the path for these enterprises to achieve profitability. This path is relatively consistent and explains most cases (consistency = 0.79, coverage = 0.65). The second path shows that the operating mode is only a crowdfunding model, which is another path for these enterprises to achieve profitability. The consistency of this path is higher than that of the previous path (consistency = 0.86), but the number of cases explained was smaller (coverage ratio = 0.11). The consistency of the entire solution is 0.76, and the coverage rate is 0.81, which is satisfactory.

### Institutional logic of the elderly care enterprises and their performance

From the institutional logic perspective of elderly care enterprises, there is only one way to realize profits for elderly care enterprises. This path shows that social welfare logic is the path for elderly care enterprises to achieve profitability. The consistency of the entire solution is 1, and the coverage rate is 0.85, which explains several cases where elderly care enterprises are profitable, which is satisfactory.

### Performance of elderly care enterprises and their equity financing intention

When the elderly care enterprises' operating mode was a private enterprise and/or public construction and private operation, the effects of institutional logic, operating mode, and performance of the elderly care enterprises on their equity financing intention were investigated. It was found that there were three implementation paths of the elderly care enterprises' active equity financing intention. The first path showed that the elderly care enterprises were driven by commercial logic and that they were in a profitable state. At the same time, their operating mode was not public construction and private operation. These three conditions were the path for elderly care enterprises to actively seek equity financing intention. This path was relatively consistent and explained most cases of active equity financing intention of elderly care enterprises (consistency = 0.77, coverage ratio = 0.67). The second path showed that these enterprises were driven by high commercial and low social welfare logic and that they were in a profitable state. At the same time, the operating mode of the elderly care enterprises was a private enterprise. These four conditions existed at the same time because the elderly care enterprises actively had equity financing intention. The consistency of this path was higher than that of the previous path (consistency = 0.83), but the number of cases explained was relatively small (coverage ratio = 0.33). The third path showed that the elderly care enterprises were driven by low commercial and high social welfare logic. The operating mode was public construction and private operation, rather than private enterprise, and their performance was poor. This was the path for such enterprises for active equity financing intention. The agreement was high (consistency = 0.77), but only a few cases were explained (coverage = 0.07). The consistency of the whole solution was 0.87 and the coverage was 0.81, which was satisfactory.

When the operating mode of elderly care enterprises was crowdfunding, the influence of their institutional logic, operating mode, and performance on the equity financing intention was investigated. It was found that there were two ways to realize the active equity financing intention of the elderly care enterprises. The first path showed that such enterprises were driven by high commercial and low social welfare logic and that they were profitable. These three conditions were the path for elderly care enterprises for active equity financing intention. This path was relatively consistent and explained some cases of active equity financing intentions of elderly care enterprises (consistency = 0.86, coverage ratio = 0.4). The second path showed that such enterprises were driven by commercial logic and that they were in a profitable state. At the same time, their operating mode was crowdfunding. These three conditions were the path for the elderly care enterprises to have active equity financing intention. The consistency of this path was higher than that of the previous path (consistency = 0.1), but the number of cases explained was smaller (coverage ratio = 0.13). The consistency of the whole solution was 0.46 and the coverage 0.875, which was quite satisfactory.

The schemes and paths for equity financing intention of elderly care enterprises are shown in Table 2. From their operating mode, there are two ways to realize profits for elderly care enterprises. From the institutional logic perspective of elderly care enterprises, there is only one way to realize profits for elderly care enterprises.

**Table 2:** Schemes and paths for elderly care enterprises to introduce investment result conditions.

** 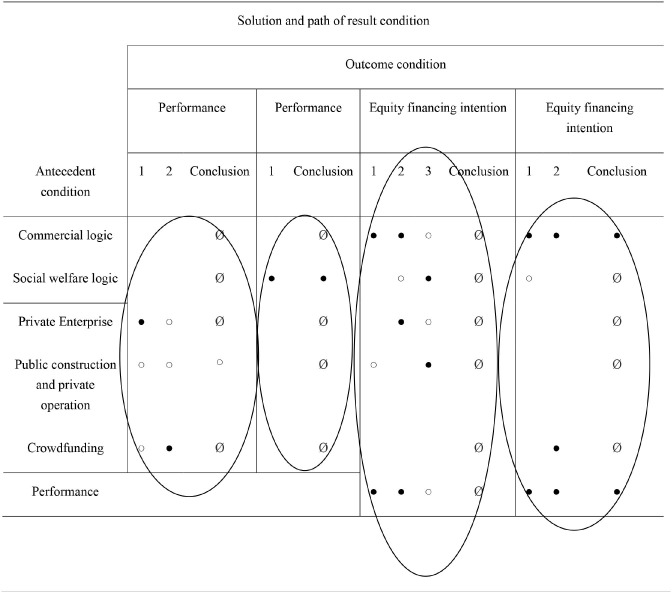 **

When the operating mode of elderly care enterprises was private enterprise and/or public construction and private operation, it was found that there were three implementation paths of active equity financing intention of elderly care enterprises. When the operating mode of elderly care enterprises was crowdfunding, it was found that there were two ways to realize the active equity financing intention of the elderly care enterprises.

## Discussion and conclusion

This study explored the public construction and private operation of elderly care enterprises. Previous studies have mostly discussed for-profit and non-profit nursing homes (6).

Regarding the impact of the operating mode on the performance of elderly care enterprises, the solution showed that non-public construction and private operation were necessary conditions for the profitability of elderly care enterprises.

Regarding the impact of institutional logic on the performance of elderly care enterprises, social welfare logic was necessary for the profitability of elderly care enterprises.

When the operating mode of elderly care enterprises was private enterprise and/or public construction and private operation, under the combined effect of institutional logic, operating mode, and performance of the elderly care enterprises on its equity financing intention, and if the influence of individual cases in path 3 was excluded, the effect was large. In most cases, commercial logic was a necessary condition for such enterprises to have active equity financing intention. In addition, high performance was also a necessary condition for elderly care enterprises to have active equity financing intention. Low social welfare logic was a sufficient condition (non-essential condition) for elderly care enterprises to have active equity financing intention. The operating mode as a private enterprise was a sufficient condition (non-essential condition) for elderly care enterprises to have active equity financing intention. At the same time, the operating mode as non-public construction and private operation was a sufficient condition (non-essential condition) for elderly care enterprises to have active equity financing intention.

Based on the relationship between business performance and equity financing intention, for-profit organizations can attract private investors, such as private equity firms, because they can pay dividends. By contrast, non-profit organizations rely on financial means such as loans, donations, or grants (19).

As per Bos et al. (6), most for-profit facilities in the Netherlands are chain-affiliated. A fifth of publicly contracted for-profit nursing homes are owned by private equity; international chains own a quarter. For-profit nursing homes are often located in affluent areas. Chinese nursing homes generally consider equity financing only after their operation becomes profitable.

Like the Netherlands, Sweden, Norway, Canada, the United Kingdom, the United States, and other European and American countries, in China, the for-profit nursing home industry has witnessed significant growth in recent years (20). It is easier for for-profit nursing home enterprises to realize standardization, scale, and chain expansion. Subsequently, their proportion will expand.

Bos et al. (6) found the problem of insufficient responsiveness of elderly care enterprises in the Netherlands. They believed that for-profit nursing homes seemed to cope better with the changing needs than non-profit nursing homes. However, in this study, nursing homes with low-profit margins without the intention of equity financing will be able to innovate more according to the needs of the elderly.

When the operating mode of the elderly care enterprises was crowdfunding, institutional logic, operating mode, and the performance of these enterprises had a combined effect on their equity financing intention. Commercial logic was necessary for the elderly care enterprises' active equity financing intention. In addition, high performance was a necessary condition. Low social welfare logic was a necessary condition for equity financing intention, a sufficient condition (non-essential condition) for elderly care enterprises to have equity financing intention. Crowdfunding for operating mode was a sufficient condition (non-essential condition) for elderly care enterprises to have an active equity financing intention.

According to the discussion, the following conclusions can be drawn:

(1) Elderly care enterprises under the public construction and private operation mode perform poorly.

The elderly care model of “public construction and private operation” refers to the government-funded infrastructure for elderly care institutions, where bidding is used to allow social organizations or service groups to perform specific management operations. It is important to note that even if non-governmental organizations or enterprises are introduced for management and operation, “public construction and private operation” elderly care institutions still cannot be profit-making. They must reflect welfare and social welfare in the price and eligibility of accommodation and housing. The subjects are mainly elderly people with difficulties in life. At the same time, the ownership of “public construction and private operation” elderly care institutions still belongs to the government. Consequently, they must accept the supervision and management of the government civil affairs department.

“Public construction and private operation” elderly care institutions must be social welfare and commercial to ensure their long-term sustainable development. It is appropriate to allow “public construction and private operation” elderly care institutions to maintain meager profits and, at the same time, strengthen policy incentives to enhance the institutions' economic strength and service capabilities. This is conducive to promoting the healthy and rapid development of “public construction and private operation“ elderly care institutions to meet more disadvantaged needs of elderly care services.

(2) Elderly care enterprises driven by social welfare logic are more likely to obtain higher performance.

In this case, most profitable elderly care enterprises focused on serving the disabled and elderly people and on the mid-end elderly care market, operating at a meager profit of 10–15%. Their feelings, that is, the social welfare logic of operating elderly care enterprises were mostly prompted by their past or current life events. Driven by social welfare logic, elderly care enterprises establish a good reputation. With word of mouth from the elderly and their families, the occupancy rate of senior care companies is relatively high, and the occupancy rate is an important guarantee for their profitability with meager profits.

(3) Elderly care enterprises driven by commercial logic are more willing to equity financing intention when they have already achieved profits.

In this case, the elderly care enterprises driven only by social welfare logic had very low-profit margins, and some were even in a state of loss. Under the guidance of the profit-seeking nature of capital, external investors have good business acumen and know ways to achieve profitability if external investment is introduced. It is likely to conflict with the founders of elderly care enterprises. This may be the reason why elderly care enterprises driven only by social welfare logic are unwilling to have the equity financing intention. Elderly care enterprises driven only by commercial logic, that is, driven by seeking profits, are more willing to introduce external investment and earn more profits with the help from investors. These enterprises are driven by commercial and social welfare logic. They may refuse external investment to realize their social welfare value if they are in a state of loss. At the same time, elderly care enterprises that have achieved profits are more willing to introduce capital, to be driven by capital and large-scale operations to provide high-quality services for the elderly.

## Shortcomings and prospects

First, the study used secondary data. Second, the study sample size was small. Future studies can increase the case enterprise, and they can bring endowment enterprise by looking into for-profit and non-profit enterprises and comparing the quality of care of the two. Finally, future research can add the endowment enterprise service model and investors' decision-making variables, such as vision, and explore its influence on the investment attitude of elderly care enterprises.

## Data availability statement

The original contributions presented in the study are included in the article/supplementary materials, further inquiries can be directed to the corresponding author/s.

## Author contributions

YZ was responsible for manuscript writing. XZ was responsible for conceptualization. DW was responsible for formal analysis, and all of us were jointly responsible for the data coding. All authors contributed to the article and approved the submitted version.

## Funding

The study supported by Hebei Natural Science Foundation (G2022210003) and Humanities, Social Science Research Projects of Colleges and Universities in Hebei Province (SQ2021162), and Humanities and Social Science Research Project of Colleges and Universities in Hebei Province (SQ201025).

## Conflict of interest

The authors declare that the research was conducted in the absence of any commercial or financial relationships that could be construed as a potential conflict of interest.

## Publisher's note

All claims expressed in this article are solely those of the authors and do not necessarily represent those of their affiliated organizations, or those of the publisher, the editors and the reviewers. Any product that may be evaluated in this article, or claim that may be made by its manufacturer, is not guaranteed or endorsed by the publisher.
